# A first-in-human clinical study of a new SP-B and SP-C enriched synthetic surfactant (CHF5633) in preterm babies with respiratory distress syndrome

**DOI:** 10.1136/archdischild-2017-312722

**Published:** 2017-05-02

**Authors:** David G Sweet, Mark A Turner, Zbyněk Straňák, Richard Plavka, Paul Clarke, Ben J Stenson, Dominique Singer, Rangmar Goelz, Laura Fabbri, Guido Varoli, Annalisa Piccinno, Debora Santoro, Christian P Speer

**Affiliations:** 1 Neonatal Unit, Royal Maternity Hospital, Belfast, Northern Ireland, UK; 2 Department of Women’s and Children’s Health, Institute of Translational Medicine, University of Liverpool, Liverpool, England, UK; 3 Department of Neonatology, Institute for the Care of Mother and Child, Prague, Czech Republic; 4 Division of Neonatology, General Faculty Hospital and 1st Faculty of Medicine, Prague, Czech Republic; 5 Neonatal Intensive Care Unit, Norfolk and Norwich University Hospitals NHS Foundation Trust, Norwich, UK; 6 Neonatal Unit, Simpson Centre for Reproductive Health, Royal Infirmary of Edinburgh, Edinburgh, Scotland, UK; 7 Division of Neonatology and Paediatric Intensive Care, University Medical Center Hamburg-Eppendorf, Hamburg, Germany; 8 Department of Neonatology, University Children’s Hospital, Tuebingen, Germany; 9 Global Clinical Development, Chiesi Farmaceutici S.p.A, Parma, Italy; 10 University Children’s Hospital, University of Würzburg, Würzburg, Germany

**Keywords:** respiratory distress syndrome, surfactant, cohort study, safety, clinical trial

## Abstract

**Objective:**

CHF5633 (Chiesi Farmaceutici S.p.A., Parma, Italy) is the first fully synthetic surfactant enriched by peptide analogues of *two* human surfactant proteins. We planned to assess safety and tolerability of CHF5633 and explore preliminary efficacy.

**Design:**

Multicentre cohort study.

**Patients:**

Forty infants from 27^+0^ to 33^+6^ weeks gestation with respiratory distress syndrome requiring fraction of inspired oxygen (FiO_2_) ≥0.35 were treated with a single dose of CHF5633 within 48 hours after birth. The first 20 received 100 mg/kg and the second 20 received 200 mg/kg.

**Outcome measures:**

Adverse events (AEs) and adverse drug reactions (ADRs) were monitored with complications of prematurity considered AEs if occurring after dosing. Systemic absorption and immunogenicity were assessed. Efficacy was assessed by change in FiO_2_ after dosing and need for poractant-alfa rescue.

**Results:**

Rapid and sustained improvements in FiO_2_ were observed in 39 (98%) infants. One responded neither to CHF5633 nor two poractant-alfa doses. A total of 79 AEs were experienced by 19 infants in the 100 mg/kg cohort and 53 AEs by 20 infants in the 200 mg/kg cohort. Most AEs were expected complications of prematurity. Two unrelated serious AEs occurred in the second cohort. One infant died of necrotising enterocolitis and another developed viral bronchiolitis after discharge. The single ADR was an episode of transient endotracheal tube obstruction following a 200 mg/kg dose. Neither systemic absorption, nor antibody development to either peptide was detected.

**Conclusions:**

Both CHF5633 doses were well tolerated and showed promising clinical efficacy profile. These encouraging data provide a basis for ongoing randomised controlled trials.

**Trial registration number:**

ClinicalTrials.gov NCT01651637.

**What is already known on this topic?**

- Randomised trials have confirmed superiority of natural, animal-derived surfactants containing proteins, over synthetic surfactants comprised of phospholipids alone.
- New generation surfactants that contain peptides mimicking effects of surfactant proteins have shown promise but are not yet widely accepted.

**What this study adds?**

- This first–in–human trial of synthetic surfactant CHF5633, containing peptide analogues of two surfactant proteins, shows that it was well tolerated without unexpected adverse effects.
- CHF5633 is similar in volume and appearance to poractant-alfa and appears to work as effectively.

## Introduction

Respiratory distress syndrome (RDS) remains a leading cause of morbidity in preterm babies.^1^ Surfactant replacement therapy has become standard of care in RDS management.^2 3^ Comparative trials show superiority of natural, animal-derived surfactants over protein-free synthetic surfactants due to the presence of surfactant proteins SP-B and SP-C.^4^A fully synthetic surfactant would have potential advantages such as no dependence on animal sources and less batch-to-batch variability.^5^ Animal experiments suggest that synthetic surfactants containing both peptides are superior to single peptide surfactants.^6^

CHF5633 is a new fully synthetic surfactant preparation consisting of phosphatidylcholine and phosphatidylglycerol, enriched by peptide analogues of both human surfactant proteins SP-B and SP-C. When suspended in saline the final phospholipid concentration is identical to that of poractant-alfa (Curosurf, Chiesi Farmaceutici S. p. A., Parma, Italy), at 80 mg/mL and a similar small dosing volume can be used. Intratracheal administration of CHF5633 to preterm newborn rabbits resulted in marked improvement in lung expansion which is no different from poractant-alfa.^7^ The structure of the peptide analogues has been modified to be resistant to oxidative injury and may improve resistance to inactivation.^8 9^ Preterm lambs with RDS treated with CHF5633 have better lung and brain injury scores than those treated with poractant-alfa.^10^ Based on these results it was anticipated that CHF5633 would be at least as effective as natural surfactants in the treatment of babies with RDS.

Surfactant treatment is normally administered as an endotracheal fluid bolus to infants. Conducting a phase I study in adults was not appropriate. Accordingly, following consultation with regulatory agencies and ethics committees, the study was designed to recruit premature neonates with ‘mild to moderate’ RDS who would be less likely to have other comorbidities and who would respond readily to rescue with other surfactants, if required.

This study aimed to investigate the safety and tolerability of intratracheal administration of CHF5633 in preterm babies. Two different doses (100 mg/kg and 200 mg/kg) were evaluated in terms of adverse events (AEs), adverse drug reactions (ADRs), haematology and biochemistry values, incidence of comorbidities, extent of systemic exposure to protein analogues and any potential immune response. Effects of CHF5633 on oxygenation, ventilatory requirements and need for rescue surfactant was assessed to explore efficacy.

## Methods

This was a first-in-human, single-escalating dose per-cohort study on administration of CHF5633. The trial was conducted in compliance with the Declaration of Helsinki and current guidelines for Good Clinical Practice after approval by regulatory authorities in each participating country and the ethical review boards for each institution and prior registration (ClinicalTrials.gov NCT01651637). Written consent was sought before birth, or soon after, giving parents the maximum time to make an informed decision before enrolment.

Infants were eligible within 48 hours after birth if born between 27^+0^ and 33^+6^ weeks’ gestation, having clinical and radiological findings of RDS, and needing fraction of inspired oxygen concentration (FiO_2_) ≥0.35 on continuous positive airways pressure (CPAP) to maintain preductal pulse oximeter oxygen saturation (SpO_2_) in the range 90%–95%. They required a normal cranial ultrasound scan and their clinician considered that surfactant was indicated. Infants were ineligible if they had already received surfactant, were already in another study, had a major congenital malformation, if there was a history of maternal drug/alcohol abuse, a clinical suspicion of pneumonia or sepsis, a 5 min Apgar score ≤3, a history of ruptured membranes of ≥3 weeks, or if seizures or pneumothoraces were detected before enrolment. The study was unusual in recruiting infants from whom, albeit for one dose, usual treatment was withheld. It was anticipated that a single-centre study would be prohibitively slow; therefore 40 babies were enrolled from 12 centres in three European countries, with careful coordination to control recruitment. The first infant was treated on 3 October 2012 and the last completed follow-up on 23 January 2015.

Two groups of 20 infants were treated. The first cohort was given 100 mg/kg of CHF5633 (1.25 mL/kg) and the second cohort 200 mg/kg (2.5 mL/kg), administered by bolus via an endotracheal tube with a short period of manual/mechanical ventilation. No infant could receive more than one dose of CH5633. Failure of response was defined as fall in FiO_2_ <0.10 to maintain SpO_2_90%–95% within an hour after treatment. Treatment failures were rescued with either 100 mg/kg or 200 mg/kg poractant-alfa (Curosurf, Chiesi Farmaceutici, Parma, Italy). All infants could receive further doses of poractant-alfa as necessary. Decisions around premedication for intubation, positioning for surfactant administration, modes and duration of ventilatory support as well as weaning protocols were left to individual participating centres.

### Safety and tolerability

A Safety Monitoring Board (SMB) was established comprising the principal investigator from each site and an independent neonatologist. The SMB reviewed the safety profile of CHF5633 in the week following administration and provided authorisation to continue. The first four babies in each cohort were recruited individually and recruitment stopped until progress to 7 days was reviewed. The subsequent 16 babies in each cohort were recruited in groups of four before SMB review. Safety and efficacy assessments were performed in the 24 hours following CHF5633 administration (at 0.5 hour, 1 hours, 3 hours, 6 hours, 12 hours and 24 hours), in the following 6 days (at days 2, 3 and 7) and in the follow-up period (at days 10 and 28, and at 36 weeks’ postmenstrual age). FiO_2_, SpO_2_, ventilator settings and blood pressure were monitored. Haematological and biochemical indices were collected at baseline, 24 hours and between 5 days and 10 days postdose. Data on all predefined expected neonatal comorbidities and deviations from expected normal values in haematological/biochemical indices were recorded as AEs and reviewed by the SMB for expectedness, severity and potential relatedness to study medication.

### Evidence of systemic absorption and immunogenicity

Blood concentrations of SP-B and SP-C analogues were measured before, and 3 hours and 24 hours post-treatment using dried blood spots. SP-C concentrations were determined using validated HPLC-MS/MS methods (Accelera, Milan, Italy). Immunogenicity was assessed using 1 mL blood obtained 4–12 weeks after CHF5633 administration. IgG antibodies to peptides were assayed by titration versus positive control (SGS Life Science Services, Wavre, Belgium).

### Efficacy

Efficacy was evaluated by examining response to CHF5633 in terms of changes in SpO_2_, FiO_2_, mean airway pressure (MAP), peak inspiratory pressure if ventilated and positive end-expiratory pressure at specified time points. Duration of mechanical ventilation was defined as time until first extubation lasting >24 hours. Durations of CPAP and supplemental oxygen were recorded. Bronchopulmonary dysplasia (BPD) was defined as need for supplemental oxygen to maintain SpO_2_ ≥90% at 36 weeks’ postmenstrual age. The number of non-responders requiring rescue surfactant was recorded.

### Statistical analysis

Because of the exploratory nature of this study, no formal power calculation was performed. Twenty babies in each cohort were deemed sufficient for reaching useful preliminary conclusions. Categorical variables are described using summary statistics, frequency count and percentages. Continuous variables are summarised using mean, SD, or median, IQR as appropriate.

## Results

**Figure 1:**
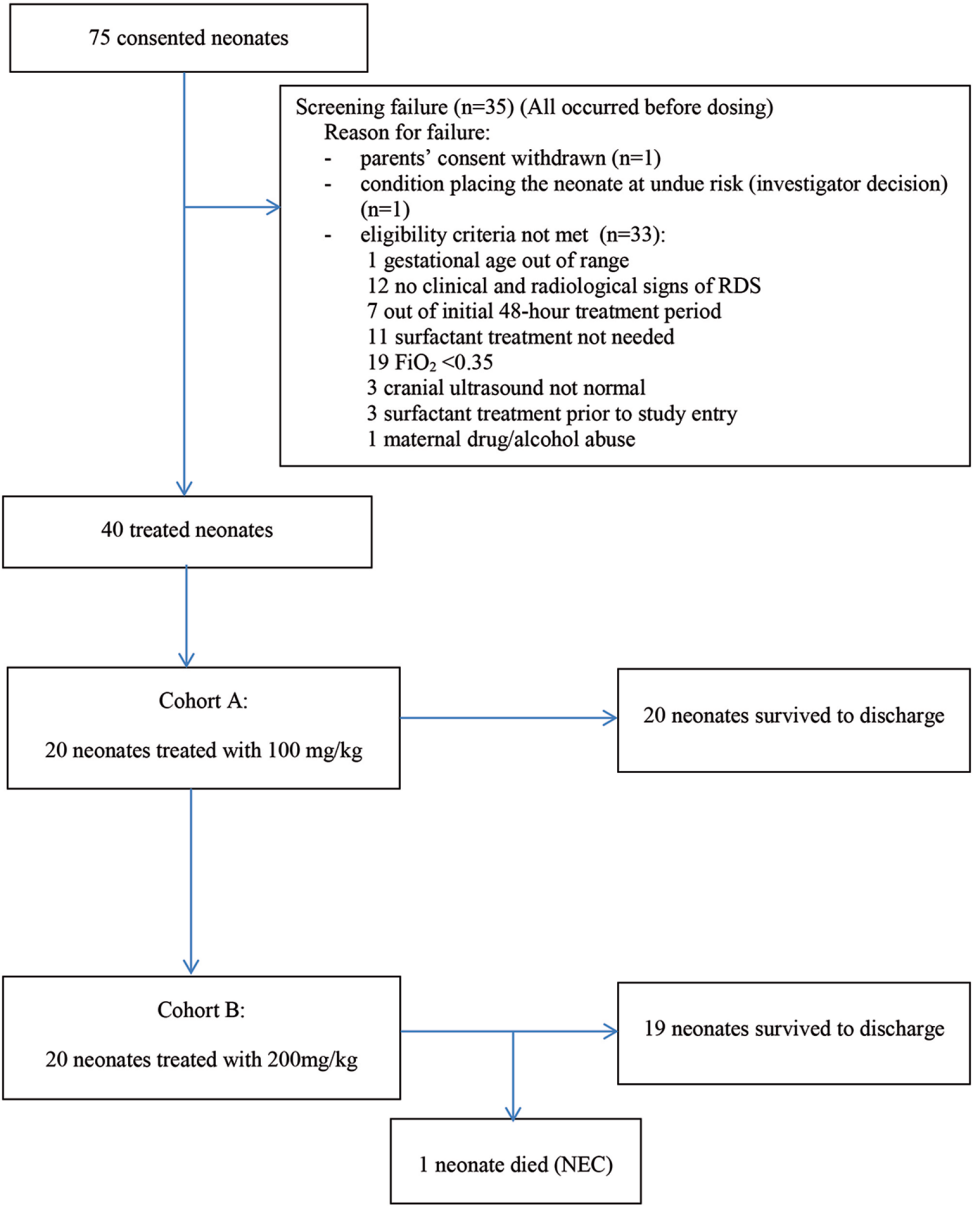
Patients’ disposition. FiO_2_, fraction of inspired oxygen; NEC, necrotising enterocolitis; RDS, respiratory distress syndrome.

A total of 75 babies were consented and 40 were dosed between October 2012 and November 2014 (figure 1). Baseline demographic data are shown in table 1. Date of patient recruitment, centre, AEs, and outcomes for each participating infant are shown in table 2.

**Table 1:** Baseline characteristics

	**100 mg/kg cohort** **(n=20)**	**200 mg/kg cohort** **(n=20)**
Gestational age (weeks)	29.6 (2.0)	29.6 (1.9)
Birth weight (g)	1274 (398)	1364 (416)
5 min Apgar	8.5 (8–9.5)	8 (7–8.5)
Gender male	11 (55%)	10 (50%)
Antenatal steroids	18 (90%)	19 (95%)
Antenatal antibiotics	11 (55%)	9 (45%)
FiO_2_ predose	0.47 (0.16)	0.52 (0.13)
Time to treatment (hours)	7 (4–23)	5 (3–16.5)

Data are shown as mean (SD) and n (%),

Median (IQR) is reported for Apgar score and time to treatment.

FiO_2_, fraction of inspired oxygen.

**Table 2:** Patient sequence, individual characteristics, adverse events and outcomes

**Treatment date**	**Country**	**Age R_x_ (hours)**	**FiO_2_**	**Sex**	**BW (g)**	**GA (week)**	**Laboratory abnormalites**	**Initial MV**	**CPAP**	**Adverse events**
**100 mg/kg cohort**										
3 October 2012	UK^2^	2	0.42	M	1505	30	↓Na ↑SBR	22 hours	6 days	
24 January 2013	UK^2^	24	0.57	M	1160	31	↑SBR	5 hours	5 days	
16 February 2013	UK^1^	5	0.38	F	900	27	↓Na ↑WCC	3 hours	39 days	
1 March 2013	UK^1^	4	0.70	M	1660	31	↑Na ↓K	17 hours	3 days	
19 April 2013	UK^1^	4	0.40	F	1010	28	↓Na	INSURE	11 days	
26 April 2013	UK^1^	24	0.45	F	1270	30	None	21 hours	none	
21 May 2013	GER^8^	8	0.44	M	805	28	↓WCC ↑SBR ↑BG	18 hours	47 days	
11 June 2013	UK^1^	6	0.40	F	1243	31	↓Na ↓K	INSURE	9 days	
27 June 2013	GER^7^	15	0.45	F	988	28	↑SBR	1 day	29 days	PDA
29 June 2013	UK^6^	4	0.36	M	1100	27	None	6 hours	6 days	IVH day 5 PVL day 28.
8 July 2013	GER^10^	4	0.40	M	2250	33	↑SBR	14 hours	2 days	
31 July 2013	UK^2^	5	0.44	F	1995	32	↑CRP	17 hours	none	
20 September 2013	UK^1^	9	0.36	F	832	28	↓Na	INSURE	36 days	
22 September 2013	GER^10^	1	0.35	M	1490	32	None	16 hours	3 days	Non-responder, PTX rescue Poractant x 2
18 October 2013	GER^8^	37	0.45	F	1490	33	↑HR	4 days	6 days	SVT day 20
6 November 2013	UK^1^	3	0.68	M	1140	28	↓Na	8 hours	23 days	PDA
22 November 2013	UK^1^	41	0.40	M	843	28	↓Na ↑SBR	3 hours	55 days	
15 December 2013	GER^7^	24	1.0	M	1371	29	↑SBR	21 hours	19 days	PDA
8 January 2014	UK^1^	10	0.36	M	1580	31	↓Na ↑SBR ↓plats	INSURE	8 days	
30 January 2014	UK^5^	22	0.36	F	850	27	None	10 days	53 days	PDA second dose Poractant day 5
**200 mg/kg cohort**										
21 February 2014	GER^7^	2	0.50	F	1050	30	↑SBR	12 hours	3 days	Apnoeic episode
23 March 2014	CZE^3^	4	0.80	M	1100	27	↑SBR	10 hours	48 days	PDA
12 April 2014	UK^6^	4	0.75	M	1685	30	↓plats	19 hours	13 days	
9 May 2014	CZE^4^	3	0.50	M	1070	28	↑SBR	INSURE	4 days	Apnoeic episode
26 May 2014	CZE^4^	26	0.38	F	1800	32	None	INSURE	4 days	
27 May 2014	CZE^4^	20	0.38	F	1075	27	None	INSURE	16 days	Episode of ET tube blockage, PDA
1 June 2014	CZE^3^	2	0.60	F	1590	30	↑SBR	INSURE	5 days	
1 June 2014	CZE^3^	3	0.40	F	1490	30	↑SBR	INSURE	4 days	
25 June 2014	CZE^3^	2	0.45	M	1130	28	↑SBR ↓Na	40 mins	26 days	
26 June 2014	CZE^3^	25	0.51	M	1060	28	↑SBR↓Na	INSURE	26 days	
27 June 2014	GER^10^	5	0.60	M	975	28	None	14 hours	12 days	PDA, PTX, second dose Poractant day 2, NEC day 13 - died
28 July 2014	UK	5	0.63	M	1690	33	None	20 hours	1 day	
29 August 2014	UK^5^	5	0.55	F	870	27	None	INSURE	33 days	
15 Septembert 2014	CZE^4^	33	0.70	F	2080	32	↑SBR	INSURE	4 days	
26 September 2014	UK^1^	13	0.36	F	876	31	↑SBR, ↓Na	INSURE	8 days	
26 September 2014	CZE^3^	10	0.50	M	1306	30	None	1 hour	12 days	
21 October 2014	CZE^3^	2	0.55	M	1720	31	↑SBR	1 hour	7 days	
11 November 2014	UK^1^	29	0.41	M	1688	30	None	23 hours	7 days	
18 November 2014	UK^6^	5	0.48	F	2190	32	None	5 hours	2 days	
21 November 2014	UK^5^	6	0.41	F	840	28	None	3 days	41 days	

↓Na, hyponatraemia; ↓K, hypokalaemia; ↑SBR, hyperbilirubinaemia; ↑WCC, leucocytosis; ↓WCC, leucopenia; ↑BG, hyperglycaemia; ↑CRP, elevated C reactive protein; ↓plats, thrombocytopenia; ↑HR, tachycardia; PTX, pneumothorax; IVH, intraventricular haemorrhage; NEC, necrotising enterocolitis; PDA, patent ductus arteriosus; PVL, periventricular leucomalacia; SVT, supraventricular tachycardia; Column 2 shows sequence of recruitment by country and site. GER-Germany; CZE-Czech Republic. Site number according to instutions of authors. Age R_x_, treatment age; FiO_2,_ fraction of inspired oxygen required just prior to dosing; BW, birthweight; GA, gestational age; MV, mechanical ventilation; CPAP, continuous positive airways pressure; ET, endotracheal; INSURE, INtubation-SURfactant_Extubation

**Figure 2:**
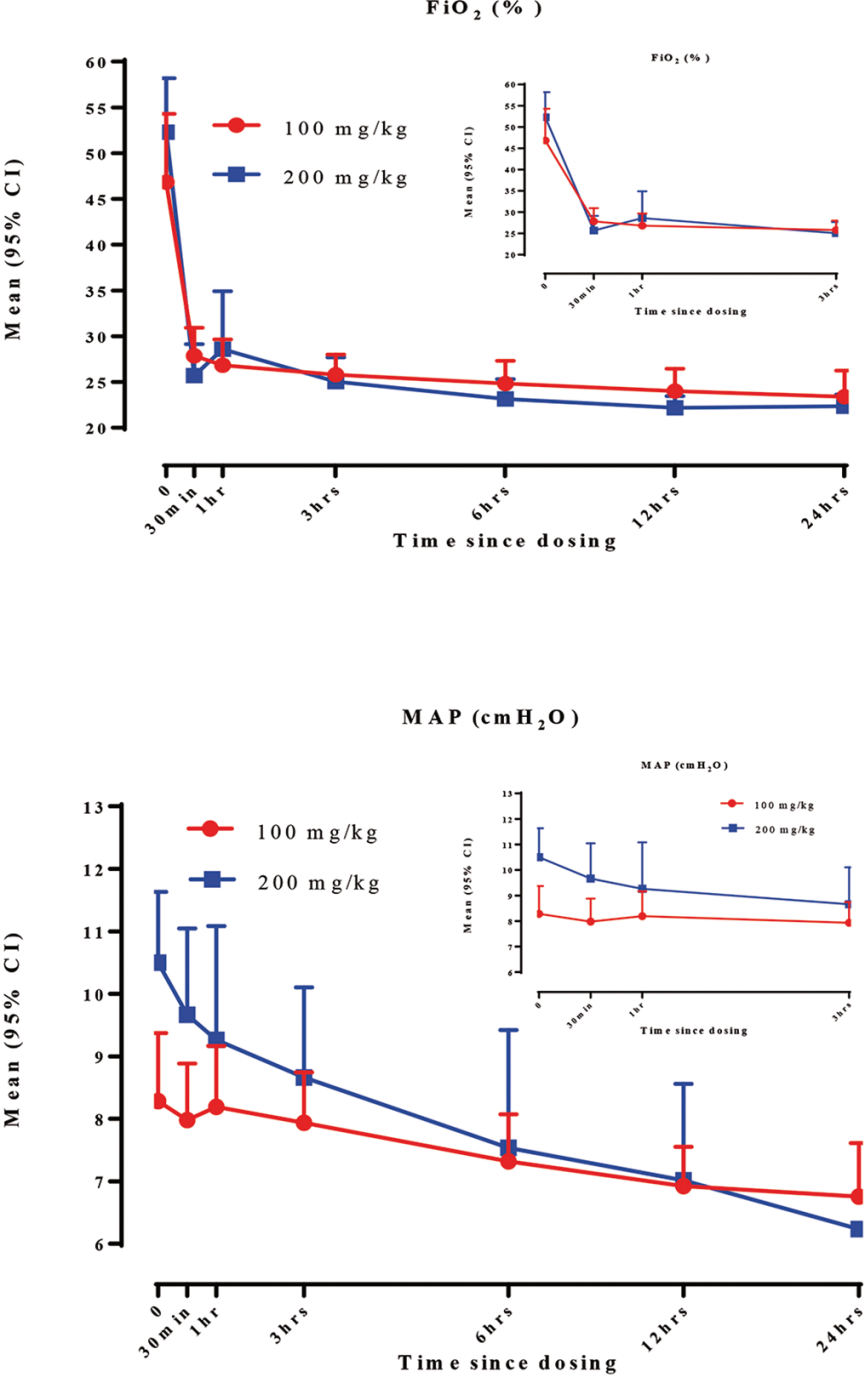
Fraction of inspired oxygen (FiO_2_) in all babies and corresponding mean airway pressure (MAP) in those undergoing mechanical ventilation 24 hours after CHF5633 in the two dosing cohorts. Bars represent SD. Data offset slightly to improve clarity. Inset shows same data over first 3 hours to illustrate speed of onset of action of effect.

Treatment with either dose of CHF5633 resulted in a rapid improvement in oxygenation with corresponding decrease in the need for supplemental oxygen and a reduced MAP (figure 2). Ten babies were extubated to CPAP immediately following CHF5633 administration and never ventilated. A further four were ventilated for <30 min. The median (IQR) duration of mechanical ventilation was 0.70 (0.30–0.91) days in the 100 mg/kg cohort and 0.30 (0.02–0.95) days in the 200 mg/kg cohort. The median (range) duration of CPAP was 14.4 (4.9–29.9) days in the 100 mg/kg cohort and 6.7 (4.0–14.1) in the 200 mg/kg cohort. There was only one case of failure to respond to CHF5633, in the first cohort. This 32-week gestation 1490 g baby was treated at 37 hours of age, and had two further 200 mg/kg doses of poractant-alfa, but still without improvement in oxygenation. A pneumothorax was diagnosed 5 hours after study treatment; this was drained and the infant responded to high-frequency oscillation. In 2 of the 40 infants a repeat dose of poractant-alfa was required as part of ongoing management (table 2). Four babies developed BPD, two from each dosing cohort.

### Systemic absorption/immunogenicity

No quantifiable concentrations of the SP-C analogue were detected in any blood sample at any time point. It was impossible to assess absorption of the SP-B analogue because in low quantities it is difficult to measure. No immune response antibodies were detected to either peptide from 36 available samples.

#### Adverse events

In total, 132 AEs were recorded, 79 experienced by 19 (95%) infants in the 100 mg/kg cohort and 53 by 20 (100%) infants in the 200 mg/kg cohort. Most events were expected clinical problems of preterm infants such as mild hyponatraemia. The investigators assessed and classified the laboratory values as normal, abnormal/not significant or abnormal/clinically significant. Most abnormalities were assessed as abnormal/not significant. Comorbidities before and after treatment are summarised in table 3 and are typical of issues in preterm babies.

**Table 3:** Comorbidities and complications of prematurity

	**100 mg/kg cohort (n=20)**		**200 mg/kg cohort (n=20)**	
	**Before n (%)**	**After n (%)**	**Before n (%)**	**After n (%)**
Any comorbidity	5 (25)	15 (75)	2 (10)	13 (65)
Anaemia	0	0	1 (5)	0
Tachycardia	1 (5)	0	0	0
Patent ductus arteriosus	1 (5)	4 (20)	0	2 (10)
Bacterial sepsis	1 (5)	1 (5)	0	0
Sepsis unspecified	0	1 (5)	0	2 (10)
Low fibrinogen	0	0	1 (5)	0
Hyperglycaemia	1 (5)	2 (10)	0	0
Hypoglycaemia	1 (5)	1 (5)	1 (5)	0
Hypoalbuminaemia	0	0	1 (5)	0
Hyponatraemia	1 (5)	8 (40)	0	3 (15)
Necrotising enterocolitis	0	1 (5)	0	1 (5)
Hyperbilirubinaemia	0	5 (25)	0	10 (50)
Intraventricular haemorrhage	0	1 (5)	0	0
Cerebral haemorrhage	0	1 (5)	0	0
Periventricular leukomalacia	0	1 (5)	0	0
Bronchopulmonary dysplasia	NA	2 (10)	NA	2 (10)
Pneumothorax	0	1 (5)	0	1 (5)
Pulmonary interstitial emphysema	0	0	0	1 (5)

NA, not applicable.

#### Adverse drug reactions

Only one ADR was reported. Following administration of 200 mg/kg to a 27^+2^ week, 1075 g infant there was temporary obstruction of the endotracheal tube for 10–15 s, which resolved quickly with no clinical consequences. The baby was extubated after 4 min, with a transient rise in FiO_2_ to 80% but reducing over the following 3 hours on CPAP, and had echocardiographic evidence of transient pulmonary hypertension. Neither allergic reactions, nor any other events potentially caused by the drug, were reported.

#### Serious adverse events

Two SAEs were reported, both occurring in the 200 mg/kg cohort: an episode of fulminant necrotising enterocolitis occurring 13 days after CHF5633 in an infant of 28 weeks’ gestation who died at 21 days, and an episode of postdischarge viral bronchiolitis considered as serious due to need for rehospitalisation. Neither SAE was considered related to the study drug.

## Discussion

This first-in-human study shows that a CHF5633 dose of either 100 mg/kg or 200 mg/kg was well tolerated, without detectable systemic absorption, and resulted in prompt and sustained improvements in respiratory function. CHF5633 is the first synthetic surfactant to contain analogues of both SP-B and SP-C. It was developed to be similar to poractant-alfa (Curosurf) in terms of its low dose volume, appearance and simple handling requirements. It requires refrigeration, and only a short period of warming in the hand prior to administration, and the volume to deliver a 200 mg/kg dose is 2.5 mL/kg. Following a single intratracheal dose the brisk response allowed rapid extubation, including the use of the INtubation-SURfactant-Extubation (INSURE) approach that is widely used with animal-derived surfactants.^11^ Apart from one patient, all infants showed an immediate clinical response with a single dose.

The population selected for this study was reasonably stable babies with RDS, deliberately chosen because of the relatively low risk of complications to allow an informative safety and tolerability assessment. They required surfactant, but were not so unwell that there was insufficient time to obtain consent and baseline investigations. Most were stable on CPAP, but with increasing oxygen requirements. Such babies are scarce, therefore recruitment at multiple sites was needed to achieve the required study population, even though this would be considered unusual for a phase I trial. The initial requirement to halt after each enrolled subject made recruitment slow.

Despite careful selection of subjects, the majority still developed a range of comorbidities that needed to be analysed within the context of what would normally be expected in a preterm baby requiring surfactant. Only one death occurred; a case of NEC considered a consequence of prematurity and unrelated to CHF5633 treatment. The single episode of transient tube obstruction was also considered a well recognised complication of surfactant therapy. Neither allergic reactions nor other events likely caused by the drug were reported. Lack of systemic exposure and of specific immune response was also reassuring. The overall rate of mortality, BPD, and their combination was low as would be expected with this selected, relatively low-risk, preterm population.^12^ These data are promising and randomised controlled trials should now determine how CHF5633 performs in a larger population including less mature and sicker infants (ClinTrials.gov NCT02452476).

Baseline characteristics were similar in the two dosing cohorts, although the predose FiO_2_ and MAP were slightly higher in the second cohort, perhaps reflecting increasing confidence at recruiting sicker babies. Statistical comparisons were not made between dosing cohorts for this reason. Both doses were efficacious, resulting in sustained improvements in oxygenation that occurred immediately after instillation. A median FiO_2_ of 0.21 was achieved within the first 24 hours of treatment. In terms of respiratory support, a shorter duration of non-invasive ventilation was found in the 200 mg/kg cohort despite them being slightly worse at baseline. This might reflect a greater improvement of lung mechanics with higher doses of CHF5633, although this needs to be tested in randomised trials.

Only one other protein-containing synthetic surfactant, lucinactant, had reached the stage of being used in comparative clinical trials in preterm neonates.^13 14^ Lucinactant contains a high concentration of the synthetic peptide sinapultide (KL-4), designed to have similar activity to SP-B, but no SP-C peptide. Lucinactant is a viscous fluid requiring warming to 44°C then vigorous shaking until it becomes a free-flowing suspension. The approved treatment dose volume is 5.8 mL/kg. In contrast, CHF5633 is more akin to poractant-alfa in terms of its handling requirements and the observed clinical response parallels that observed in trials of existing animal-derived surfactants.^15 16^

Current thinking about optimal management of RDS is to aim where possible to avoid mechanical ventilation.^17^ Administering surfactant without mechanical ventilation is gaining acceptance as a strategy to minimise lung injury.^18 19^ Fourteen babies in this study were extubated within 30 min of CHF5633 administration, including 10 where clinicians employed the INSURE technique. Future comparative trials of CHF5633 should therefore explore all potential modes of administration including minimally invasive methods.

Animal-derived surfactants require pooling of material from multiple animals. Quality control is stringent, but many stakeholders would be reassured if the theoretical risks of infection could be avoided. There is a drive towards ensuring that children of all ages have access to age-appropriate formulations. This involves tailoring administration to the needs of the child and optimising pharmaceutical quality of the product. By ensuring that the volume to be administered is small and avoiding use of animal products, the development of CHF5633 addresses these needs. Ideally it would also prove to be more efficacious in some circumstances. Studies in a sheep model of acute lung injury suggest that CHF5633 may be more resistant to inactivation than poractant-alfa.^20^ This raises the possibility that it may have advantages in severe disease, or in other causes of respiratory failure associated with surfactant inhibition.^21–24^

In conclusion, CHF5633 is the first synthetic surfactant to contain analogues to both surfactant proteins, SP-B and SP-C. This first-in-human study shows that it was well tolerated by preterm babies with moderate RDS and raised no safety concerns, with a promising clinical efficacy profile. Larger trials are warranted and if these produce similar results it is likely that this will herald a new era of synthetic surfactant treatment.
